# Can Genetic Testing Coupled with Enhanced Dopaminergic Activation Reduce Recidivism Rates in the Workers Compensation Legacy Cases?

**DOI:** 10.4172/2329-6488.1000161

**Published:** 2014-05-29

**Authors:** Kenneth Blum, Marlene Oscar-Berman, Seth H Blum, Margaret A Madigan, Roger L Waite, Thomas McLaughlin, Debmalya Barh

**Affiliations:** 1 Department of Psychiatry & McKnight Brain Institute, University of Florida College of Medicine, Gainesville, Fl., USA; 2 Human Integrated Services Unit University of Vermont Center for Clinical & Translational Science, College of Medicine, Burlington, VT, USA; 3 Department of Addiction Research & Therapy, Malibu Beach Recovery Center, Malibu Beach, CA, USA; 4 Dominion Diagnostics, LLC, North Kingstown, RI, USA; 5 Institute of Integrative Omics and Applied Biotechnology, Nonakuri, Purba Medinipur, West Bengal, India; 6 IGENE, LLC, Austin, Texas, USA; 7 Departments of Psychiatry, Neurology, and Anatomy & Neurobiology, Boston University School of Medicine, and Boston VA Healthcare System, Boston, MA, 02118, USA; 8 Department of Nutrigenomics, RDSolutions, LLC, La Jolla, CA, USA; 9 Center for Psychiatric Medicine, North Andover, Massachusetts, USA

An overwhelming segment of the world’s population possesses certain genetic variations that increase risk for genetic predispositions to substance seeking and even behavioral addictions (e.g. gambling, internet gaming, multiple sex partners etc.) that preclude them from reaching their optimum health potential, contribute to impaired health, and/or can cause involuntary indulgence in detrimental and self-destructive behaviors. This is especially true when this genetic reward deficiency (RDS) [1] problem leads to not only compulsions and excessive cravings but also impaired decision making. This brain hard wiring will ultimately lead to unwanted narcotic addictions; anti-social behavior; crime and unnecessary medical procedures that burden society [2].

It is our notion that the real genesis of all behavior, whether so-called normal or abnormal behavior, derives from an individual’s genetic makeup at birth involving gene variations that make up about 3% of the human genome. While people are not doomed because of carrying these so called variations (called alleles) this risk predisposition, due to multiple gene combinations and polymorphisms (gene variations), is expressed differently based on numerous environmental (epigenetic) elements. So carrying these known gene variants especially in those genes known to control what is called the “Brain Reward Cascade (BRC) influencing how the brain programs feelings of well-being, mediate the actual expression of these important genes [3].

It is well-established that psychiatric disorders are complex multifactorial illnesses involving chronic alterations in the brain reward circuitry. While it is agreed that genetic factors are important in the etiology of disorders such as RDS, relatively high rates of discordance among identical twins suggest the importance of other mechanisms. Certainly, environmental factors such as stress or prior drug exposure play a role in the onset of these illnesses. There is evidence that sustained abnormalities are maintained by epigenetic modifications in specific brain regions. Addictive behavior can be modeled in animals by inducing disease-like states through environmental manipulations (e.g., chronic stress, drug administration) [3].

As David Smith the founder of the Haight-Ashbury Clinic stated “Love Needs Care” and that is what positive caring has in common with powerful epigenetic effects. We firmly embrace this “love and care” concept as part of any treatment goal with the knowledge that through epigenetic effects either methylation and or deacetylation gene expression will lead to positive dopamine release at the reward centers of the brain [4]. Understanding the importance of epigenetics and its effects on chromatin structure will lead to new therapeutic targets to combat for example drug seeking behavior.

In fact, we promote the concept that the core of predisposition to these behaviors is a set of genes (called candidate genes), which mediate a feeling of well-being via chemical messenger (i.e. neurotransmitters) interaction at the “reward site” of the brain (N. Accumbens), leading to normal dopamine release and influencing dopamine receptor density (the actual number of dopamine receptors). A low number of Dopamine receptors suggest a hypodopaminergic function as manifested in all addictive disorders. When there are a low number of dopamine receptors, the person will be more prone to seek any substance or behavior that stimulates the dopaminergic system (a sort of “dopamine fix”). In fact, Nora Volkow the current director of the National Institutes of Drug Abuse (NIDA) stated that “all roads lead to dopamine” further described by Blum et al. [5].

To understand generalized craving behavior, due to hypodopaminergic function (a deficiency of reward responsiveness [blunted], individuals self-medicate through biochemical (illicit or non-illicit) attempts to alleviate or compensate for the low dopaminergic brain activity via drug-receptor activation (alcohol, heroin, cocaine, glucose, etc.) [6]. This will substitute for the lack of reward and yield a temporary compensatory sense of well-being. It is this low Dopamine genetic variant that sets these so called “Legacy Case” workers (see below) up for a predisposition for addiction. A very high percentage of these workers are involved in the “injury-treatment-medication-injury” revolving door cycle and may even carry genetic variants (serotonergic and dopaminergic) that result in a higher incident of accidents (e.g. driver accident tendentiousness) for these prone individuals [7].

In order to help explain this so called pseudo self-healing process, we are cognizant of known dysfunctional diagnosis known as “Legacy Cases.” These cases are a major problem within the Workers Compensation insurance carriers system. They are very similar to another dysfunctional diagnose such as Fibromyalgia [8]. Legacy cases are patients with chronic pain that have a monthly narcotic cost of well-over $5,000 per month and have very low functionality in terms of daily living. These claims have been through the gambit of treatments (pharmaceutical, surgical, injections and rehabilitation) with little to no positive outcomes. One reason why these patients do not respond to traditional pain treatments is that they fall into a medical category of “Hypodopaminergic” or Reward Deficiency Syndrome (RDS) [9]. A major problem is that the neuro-circuits of their brains are incapable of having so called normal function of dopamine (called Homeostasis) so they become legal iatrogenic (clinically induced by legally prescribed opioids) addicts [10]. These patients just as the Fibromyalgia patients (which also seem not to respond to traditional therapies) require Dopamine Agonist Modalities (DAM). A number of clinical studies utilizing DAM have shown significant reversal of patients’ craving behavior, reduction of AMA (Against Medical Advice) rates; relapse prevention as well enhancement of quality of life during recovery including happiness (joy) [11].

One of the major underlying problems relating to these Legacy Cases is the inappropriate misdiagnosis and non-productive treatments these patients experience because of utilization of paper-pencil tests rather than sound candidate genetic testing [12]. We strongly believe that especially in low functioning “Legacy Cases” these patients may display high risk due to multiple gene variations across the brain including areas like the Pre-Frontal Cortex Cingulate Gyrus (a site of relapse due to decision –making activity) as well the N. Accumbens (a site of craving behavior). Simply put many of the “Legacy Case” patients’ carry multiple gene variations (neurotransmitters and synthesis producing and metabolic genes) that lead to low Dopamine function (possibly reduced dopamine being released from nerve cells into the synapse (space between two nerve cells) as well as synaptic high breakdown (catabolism) [13]. Correctly, reversing this condition and participating in appropriate treatments will balance their reward brain chemistry by increasing endogenously higher dopamine levels as well as dopamine’s overall reward function. The coupling of genetic testing to determine a person’s genetic risk for addiction and DAM in conjunction with other dopamine promoting effects (i.e. exercise, yoga, electrotherapy, cryotherapy, 12 steps, etc.) will result in pain reduction [14].

Our goal in terms of assisting insurance carriers is to reduce iatrogenic induced opioid addiction by reducing subsequent dependence on these powerful opiates such as even Suboxone thereby promoting “Legacy Case” closure [15]. Closure of these difficult cases will not only benefit the patient’s relief of opioid dependence potentially reducing the “revolving door” phenomena, but will help these insurance carriers to return financial reserves held in abeyance back to the carrier’s general fund. Moreover, utilization of the Genetic Addiction Risk Score (GARS_Rx_™) especially at the portal of entry of patients presenting with either acute or chronic pain will identify patients with high RDS risk and as such will flag these patients’ with genetic knowledge and attenuation of over-prescribed powerful pain reliving opiates at inception of their treatment and physician promotion of subsequent alternatives for pain control (electrotherapy, acupuncture etc.) [16]. Moreover, knowing what the genetic variants are within the BRC will assist the physician in correctly prescribing an effective treatment plan for a better prognosis.

## Conclusion

To reiterate utilizing a new paradigm shift in both genetic testing and enhancing functional connectivity of the brain with DAM should improve the health and well-being of the “Legacy Claim” patients because the major underlying root causes of opiate dependency will be reduced. It will additionally curb abuse within the Workers’ Compensation Program and provide a significant monthly cost savings to the carrier and employer. In fact utilizing our approach in a hypodopaminergic genetically tested patient with GARS, having a workers Comp payment for medications over $50,000 per month has been successfully treated with DAM as well as other natural modalities, being opioid free for over two years [17]. Our main mission is to “redeeming joy”™ while inducing higher functionality leading to returning to the workforce. Following additional required research and further confirmation cautious interpretation is encouraged.
